# Roles and therapeutic potential of CD1d-Restricted NKT cells in inflammatory skin diseases

**DOI:** 10.3389/fimmu.2022.979370

**Published:** 2022-09-02

**Authors:** Sung Won Lee, Hyun Jung Park, Luc Van Kaer, Seokmann Hong

**Affiliations:** ^1^ Department of Integrative Bioscience and Biotechnology, Institute of Anticancer Medicine Development, Sejong University, Seoul, South Korea; ^2^ Department of Pathology, Microbiology and Immunology, Vanderbilt University School of Medicine, Nashville, TN, United States

**Keywords:** CD1d-restricted NKT cells, glycolipid antigens, atopic dermatitis, allergic contact dermatitis, psoriasis, UV-induced skin inflammation

## Abstract

Natural killer T (NKT) cells are innate-like T lymphocytes that recognize glycolipid antigens rather than peptides. Due to their immunoregulatory properties, extensive work has been done to elucidate the immune functions of NKT cells in various immune contexts such as autoimmunity for more than two decades. In addition, as research on barrier immunity such as the mucosa-associated lymphoid tissue has flourished in recent years, the role of NKT cells to immunity in the skin has attracted substantial attention. Here, we review the contributions of NKT cells to regulating skin inflammation and discuss the factors that can modulate the functions of NKT cells in inflammatory skin diseases such as atopic dermatitis. This mini-review article will mainly focus on CD1d-dependent NKT cells and their therapeutic potential in skin-related immune diseases.

## Introduction

Inflammatory immune responses in the skin are attributed to exposure to allergic irritants (e.g., metals, fragrance chemicals, preservatives, antibiotics, and drugs), pathogens (e.g., *Staphylococcus aureus* and fungi), and ultraviolet (UV) radiation (1, 2). While many immune cell types contribute to the pathogenesis of inflammatory skin diseases, we focus here on the role of natural killer T (NKT) cells, a subset of innate-like T cells that co-express T and NK cell receptors. In general, NKT cells recognize glycolipid antigens presented by MHC I-like CD1d molecules. NKT cells can be further classified into two subsets based on their distinct TCR characteristics: type I (invariant TCRα chain, Vα14Jα18 in mice and Vα24Jα18 in humans) and type II (diverse TCR, non-Vα14Jα18/Vα24Jα18) NKT cells (3–6). Type I NKT cells are also called invariant NKT (iNKT) cells, owing to their unique expression of an invariant TCR alpha chain, and these cells react with the prototypical glycolipid antigen α-galactosylceramide (α-GalCer). Both subsets of NKT cells make crucial contributions to skin inflammatory responses, playing either protective or pathogenic roles in animal models of inflammatory skin disorders (7, 8). Consistent with these animal studies, patients with inflammatory skin diseases (e.g., atopic dermatitis (AD), allergic contact dermatitis (ACD), psoriasis, and UV-induced skin inflammation) display functional alterations in CD1d-restricted NKT cells (7, 8). Functional heterogeneity of CD1d-restricted NKT cells may also contribute to the distinct outcome of various skin diseases. In particular, depending on the expression profile of CD4 and CD8 co-receptors, type I NKT cells can be subdivided into CD4^+^ and CD4^-^CD8^-^ (double negative, DN) subsets. Furthermore, type I NKT cells are functionally subclassified by differential expression of transcription factors: T-bet for NKT1, GATA3 and PLZF for NKT2, and RORγt for NKT17 cells (3–6). This mini-review will discuss the immunomodulatory roles of CD1d-restricted NKT cells in various inflammatory skin disorders.

## Atopic dermatitis (AD)

AD is a pruritic and chronic inflammatory skin disorder characterized by T helper type 2 (Th2)-dominant responses. It is elicited by pro-Th2 cytokines (e.g., thymic stromal lymphopoietin (TSLP), IL25, and IL33) released by keratinocytes and fibroblasts (9). Interestingly, AD’s pathogenesis in humans closely correlates with quantitative and qualitative changes in iNKT cells among peripheral blood mononuclear cells (PBMCs) (10–17). Recently, several studies have reported that AD patients display phenotypic changes in CD1d-restricted NKT cells, suggesting their potential role in AD pathogenesis.

The frequencies of surface immune cell markers [i.e., CD4/CD8 (10, 12, 14, 15), CD161 (13), and CXCR4 (17)] among NKT cells of AD patients are altered. In addition, one study reported that AD patient-derived IgG antibodies induce selective expansion of the CD4^+^ subpopulation in thymic but not splenic iNKT cells from non-atopic infants and such IgG-stimulated CD4^+^ iNKT cells produced high amounts of IL4, IL17, and IL10 (18). Recently, Sun et al. reported that skin-resident CXCR4^+^ iNKT cells recruited by fibroblast-derived CXCL12 aggravate AD through excessive secretion of both IFNγ and IL4 (17). Conversely, our study demonstrated that adoptive transfer of iNKT cells (mostly DN cells) from Vα14 TCR transgenic (Tg) NC/Nga (NC) mice effectively prevented spontaneous AD development in recipient NC mice by increasing IFNγ-producing CD8^+^ T cells and regulatory T (Treg) cells (19). Furthermore, consistent with our report, previous studies have shown that DN iNKT cells can protect against airway hypersensitivity in a mouse model of asthma *via* expansion of Treg cells (20, 21). Moreover, based on studies that influenza infection or injection of Th1 cytokine-biasing glycolipids (e.g., α-C-GalCer and napthylurea-modified α-GalCer) during the neonatal period can induce preferential expansion of DN NKT cells in mice, expansion of DN NKT cells during early life might be effective in preventing AD development (20, 21). However, repeated injection of α-GalCer into Vα14 TCR Tg NC mice exacerbated AD pathogenesis, indicating that Th2-biased iNKT cells induced by repeated α-GalCer injection exhibit adverse effects on AD symptoms (22). This study therefore suggests that continuous exposure to pathogen-derived glycolipid antigens can dramatically influence AD development.

Pro-Th2 cytokines, including TSLP, IL33, and IL25, play a critical role in initiating Th2 immune responses in AD (9). It has been reported that enhanced expression of keratinocyte-derived TSLP in AD patients activates iNKT cells to secrete IL4 and IL13, which positively correlated with AD severity (23). Moreover, murine keratin-14^+^ keratinocytes and HMGB1^+^ fibroblasts in the skin express high levels of IL33 after intradermal injection of *S. aureus* (24). Although IL33- and IL25-induced iNKT cell activation has been shown to play an essential role in a mouse model of asthma (25, 26), it remains unclear whether CD1d-restricted NKT cells stimulated by IL33 and IL25 contribute to AD progression. It has been reported that the skin lesions of most AD patients are heavily colonized with *S. aureus* (27). In particular, the prevalence of multi-drug resistant *S. aureus* (MRSA) in children with AD has continued to increase for over ten years (28). Unlike α-GalCer, heat-killed *S. aureus* induces the secretion of substantial amounts of IFNγ rather than IL4 by iNKT cells *via* CD1d-dependent activation in the presence of DCs (29). In addition, an *S. aureus*-derived lipid fraction, containing a 60:40 ratio of PG (phosphatidylglycerol):lysyl-PG, stimulated type II NKT cells through CD1d-TCR engagement to produce IFNγ, resulting in protection against MRSA infection (30). However, treatment with sulfatide, a well-known endogenous ligand for type II NKT cells, significantly attenuated *S. aureus* sepsis *via* decreased secretion of TNFα and IL6 cytokines in the blood (31), suggesting that type II NKT cells might be involved in regulating *S. aureus* pathogenesis in the skin.

Epicutaneous and intradermal infection of *S. aureus* induces skin inflammation through MyD88-dependent signaling (32). Additionally, TLR-activated DCs can present self-lipid antigens (e.g., β-D-glucopyranosylceramide (β-GlcCer) and iGb3) to activate iNKT cells in a MyD88-dependent fashion (33). Furthermore, rapid up-regulation of Ugcg (ceramide glucosyltransferase) in DCs accompanied by *S. aureus* infection induces endogenous β-GlcCer accumulation in DCs, resulting in the CD1d-dependent presentation of β-GlcCer to iNKT cells. Notably, β-GlcCer C24:1 was the most potent β-GlcCer variant to activate iNKT cells in TLR-stimulated DCs (34). These findings support the notion that CD1d-restricted NKT cells contribute to regulating *S. aureus* infection-elicited immune responses *via* CD1d-dependent TCR engagement. It is well established that staphylococcal superantigens (SsAgs), such as staphylococcal enterotoxin B (SEB), contribute to the pathogenesis of skin inflammation in AD (35). In addition, SsAgs expand Vβ8^+^CLA (cutaneous lymphocyte-associated antigen)^+^ memory T cells in PBMCs and induce their infiltration into skin lesions of AD patients (36). Since iNKT cells predominantly express a Vβ8 chain paired with a Vα14-Jα18 chain, *S. aureus* (strain COL)-derived superantigen SEB directly stimulated iNKT cells to release IFNγ rather than IL4 in an MHC II- but not CD1d-dependent manner (37).

## Allergic contact dermatitis (ACD)

ACD, also called “contact hypersensitivity (CHS)”, is considered a Type IV or delayed-type hypersensitivity (DTH) because it is mainly mediated by T cells. Many iNKT cells infiltrating ACD skin lesions display an effector phenotype with high levels of IFNγ and IL4, indicating that iNKT cells might play an essential role in ACD pathogenesis. Interestingly, the ratio of two cytokines, IFNγ and IL4, in the skin of these patients appears to diverge in a manner dependent on the allergen type (38).

Nickel allergy is the most prevalent metal-induced ACD. In the murine experimental setting of nickel allergy, iNKT cells are predominant in inflamed skin. They secrete high amounts of Th1-type cytokines (i.e., TNFα, IFNγ, and IL2) as well as cytolytic molecules (NKG2D, perforin, granzymes A and B, and FasL), suggesting that iNKT cells influence nickel allergy development (39). Notably, among iNKT cells in the ACD skin lesions, the DN iNKT subpopulation is over three times more abundant than the CD4^+^ iNKT cell subset (39). Furthermore, since DN iNKT cells exhibit a Th1-like phenotype with high IFNγ and IL2 but low IL4 secretion in mice (19), and CD4^-^ iNKT cells express high levels of NKG2D on their surface in humans (40), it is likely that DN but not CD4^+^ iNKT cells play a pathogenic role in nickel allergy. Moreover, it has been reported that keratinocytes do not activate resting iNKT cells but could serve as targets for activated iNKT cells releasing cytolytic granules such as perforin and granzymes in ACD patients (41). Furthermore, the cytotoxicity of iNKT cells against keratinocytes was CD1d-dependent, consistent with a pathogenic role in ACD. However, the precise mechanism of iNKT cell activation in nickel allergy remains to be elucidated. Since TLR4 signaling (in humans) and MyD88/IL1 signaling (in mice) have been implicated in nickel-induced ACD (42–44), either immune cells (i.e., DCs and macrophages) or non-immune cells such as keratinocytes may mediate iNKT cell activation.

Different types of ACD can be induced by haptens (e.g., 2,4-dinitrofluorobenzene (DNFB), dinitrochlorobenzene (DNCB), and oxazolone). In murine DNFB-induced ACD, iNKT cells attenuate ACD pathogenesis *via* modulation of CD8^+^ T cell activation but not Treg cell induction, suggesting a protective role of iNKT cells. These effects were attributed to IL4 and IL13 released from iNKT cells stimulated by hapten-loaded DCs through a CD1d-dependent pathway (45). A protective role of iNKT cells has also been reported in the DNCB-induced ACD mouse model, in a mechanism involving suppression of IFNγ production (46). These protective effects were strongly linked with increased IL10-producing regulatory B (Breg) cells constituting most of the CD1d^hi^CD5^+^ subset in the spleen and peritoneal cavity (46). However, a previous study demonstrated that iNKT cells play pathogenic roles in the oxazolone-induced ACD murine model. Oxazolone sensitization triggers iNKT cells to produce IL4 to co-activate innate-like B1 cells along with specific antigens for IgM antibody production, ultimately exacerbating ACD by recruiting effector T cells (47). Previous studies provide support for a critical role of CD1d-dependent cognate interactions between iNKT cells and B1 cells to induce B1 cell-derived circulating IgM in oxazolone-induced ACD (48–50). Moreover, the progression of oxazolone-induced ACD could be attenuated effectively by intraperitoneal injection of the iNKT cell antagonist α-ManCer (51), which provides support for the pathogenic role of iNKT cells in this model. Taken together, iNKT cells can play differential roles in ACD depending on the type of hapten employed in disease induction.

## Psoriasis

Psoriasis is a chronic immune-mediated skin disorder characterized by red, scaly, thickened, inflamed, and itchy skin. Pro-inflammatory cytokines (i.e., TNFα, IFNγ, IL17, and IL22) are central in initiating psoriatic skin inflammation (52). Interestingly, Vα24^+^Vβ11^+^ NKT cells (53) or CD3^+^CD56^+^ NKT cells (54) in PBMCs were statistically decreased in number in psoriasis patients compared with healthy controls. In contrast, the relative frequencies of iNKT2 and iNKT17 cells in PBMC of psoriatic patients were increased compared with healthy controls, whereas total and CD69^+^ iNKT cells were significantly decreased in number (55). Moreover, infusion therapy to psoriatic patients with CD3^+^CD56^+^ NKT cells (which likely consist of CD1d-restricted NKT cells) restored CD3^+^CD56^+^ NKT cell levels in patient PBMCs, leading to improved skin lesions in severe psoriasis (56). These studies indicate that CD56^+^ NKT cells contribute to regulating psoriatic skin inflammation, possibly by producing Th2 cytokines such as IL4.

Conversely, psoriatic patients have significantly higher numbers of skin CD161^+^ NKT cells in the pre-psoriatic skin than in normal skin (57). Importantly, CD1d-restricted CD161^+^ NKT cells from psoriatic patients were capable of rapidly producing IFNγ upon recognition of glycolipid antigen presented by CD1d on keratinocytes (58). In addition, intradermal injection of these cells into pre-psoriatic human skin grafted on severe combined immunodeficiency (SCID) mice caused the development of psoriatic plaques (59). Furthermore, injection of allogeneic blood-derived psoriatic lymphocytes induced psoriatic plaques in the skin of SCID mice receiving human skin xenografts, and increased CD161^+^ NKT cell infiltration closely correlated with psoriasis pathogenesis (60). Another study also showed that CD1d-expressing keratinocytes could stimulate CD161^+^ NKT cells to produce a more significant amount of IFNγ, resulting in exaggerated psoriasis (58). In addition, increased activity of PKCζ in TNFα-stimulated keratinocytes has been implicated in enhanced Vα24 and CD1d expression in psoriatic skin (61). Collectively, these studies suggest that CD161^+^ NKT cells play a central role in the pathogenesis of psoriasis by inducing Th1-type cytokine production in a CD1d-dependent manner.

It is well known that patients with psoriasis show increased transepidermal water loss (TEWL), which reflects skin barrier abnormalities (i.e., increased permeability), accompanied by a reduction of epidermal ceramides (Cer) (62). In the upper epidermis, β-glucocerebrosidase (GlcCer’ase) was decreased in psoriatic skin compared with normal skin, suggesting that the decreased activity of GlcCer’ase may be responsible for GlcCer accumulation and a reduction of Cer in the lesional skin of psoriatic patients (63). In particular, the accumulation of 5-25% GlcCer in the stratum corneum (together with the concomitant loss of 5-25% Cer) has been implicated in increased TEWL in human skin (64). Since TNFα increases CD1d expression on keratinocytes and GlcCer-rich fractions activate NKT cells in a CD1d-dependent manner (61, 65), it will be worthwhile to investigate whether treatment with both a GlcCer’ase activator and a TNFα inhibitor (i.e., infliximab, adalimumab, or etanercept) can improve clinical symptoms by controlling pathogenic CD1d-restricted NKT cell activation in psoriatic patients.

## UV-induced skin inflammation

CD1d-dependent iNKT cells play protective roles in UV-induced skin inflammation. For example, iNKT cell-deficient CD1d KO mice are more resistant to UV-induced apoptosis of keratinocytes and fibroblasts (66). Furthermore, Fukunaga et al. demonstrated that UV irradiation suppresses DNFB-induced CHS in mice. Such immunoregulatory effects of UV exposure are associated with enhanced IL4 production by iNKT cells induced *via* CD1d-expressing Langerhans cells (LCs) in skin-draining lymph nodes (67). These studies identify CD1d-dependent NKT cells as therapeutic targets to modulate UV exposure-elicited Th1-type skin immune diseases such as CHS. Interestingly, two different NKT cell-deficient mouse models displayed distinct outcomes in response to UV-induced skin inflammation: Jα18 KO and CD1d KO mice generated pathogenic and protective responses, respectively. Although these results might reflect differential functions between type I and type II NKT cells (68), the effect of altered TCR repertoire diversity in Jα18 KO mice should be reassessed (69–71).

UVB irradiation induces the accumulation of sphingolipids such as GlcCer in the mouse epidermis (particularly the stratum corneum), resulting from markedly reduced GlcCer’ase activity, with a concomitant increase in TEWL (72). One previous study showed that *in vivo* glucosylceramide synthase (GCS)-dependent glycosphingolipid (GSL), in particular GlcCer, influences iNKT cell development in the mouse thymus (73). Because endogenous GlcCer is widely found in most mammalian tissues, the GlcCer-enriched lipid fraction could activate iNKT cells in a CD1d-dependent manner (34, 65). Therefore, these findings suggest that the CD1d-dependent immune suppressive effects of UV exposure might be attributed to iNKT cell recognition of CD1d loaded with endogenous glycolipids such as GlcCer. In addition, UV irradiation has beneficial effects on bacterial infection-induced pathology. For instance, in UV-irradiated mice, CD4^+^DX5^+^ NKT cells produce IL4 to inhibit *Candida albicans* infection-induced DTH immune responses in a CD1d-dependent manner (74).

## Other skin-related diseases

In patients with scleroderma, also known as systemic sclerosis (SSc), numerical and functional defects of iNKT cells have been identified (75). B cells have been suggested as one of the key players in SSc pathogenesis. Scleroderma patients display significantly higher IL6 production by B cells, and suppression of B cell-derived IL6 was attributed to cell contact between iNKT and CD1d-expressing B cells *via* the CD1d-TCR axis (76). Furthermore, iNKT cells have been reported to play important roles in wound healing (77). For example, after skin wound induction, the healing process was delayed in iNKT cell-deficient Jα18 KO mice, which was associated with reduced IFNγ production. iNKT cells promote skin wound healing by preventing prolonged neutrophilic inflammatory responses (78). In addition, iNKT cells promote the clearance of *Pseudomonas aeruginosa* at the wound site during skin wound healing by inducing IL22, IL23, and antimicrobial peptide S100A9 after bacterial infection (79). Alopecia areata (AA) is a skin disorder that causes hair loss. A previous study showed that, in the human skin xenograft model, IL10-secreting iNKT cells prevent AA development, suggesting that their activities are related to suppression of NKG2D^+^CD8^+^ T cells, which are potential mediators of AA (80). In addition, vitiligo patients display defective frequencies and functions of iNKT cells in PBMCs (81).

## Concluding remarks

**Figure 1:**
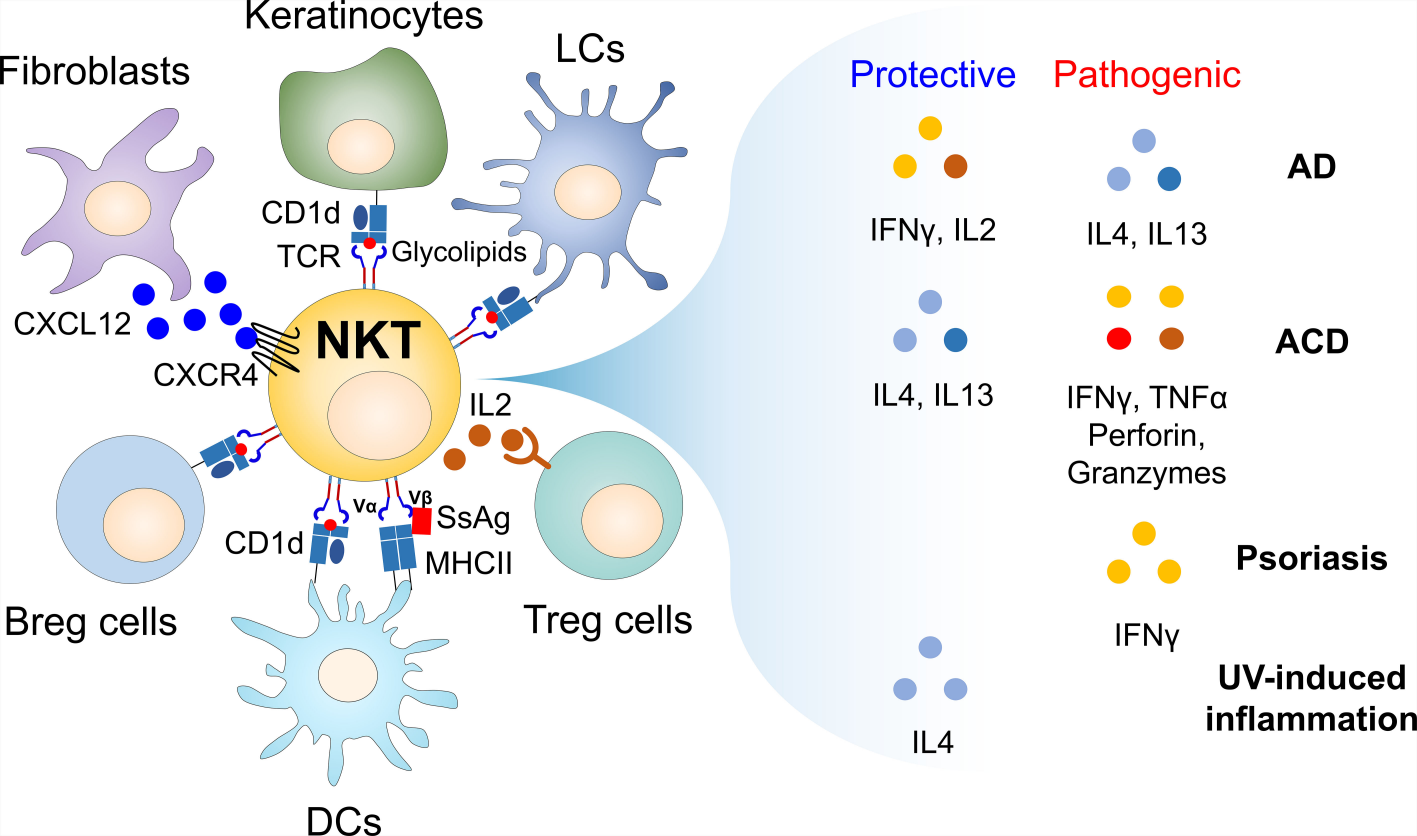
Cellular networks of CD1d-restricted NKT cells and their soluble factors in regulating skin inflammatory responses. Since the skin is constantly exposed to external stimuli such as pathogens and allergens, inflammatory immune responses occur when the skin barrier is broken. For example, during infection, endogenous glycolipids (i.e., β-GlcCer) induced by TLR signaling can stimulate CD1d-restricted NKT cells to produce large amounts of soluble factors such as cytokines that promote or regulate immune responses, contributing to maintaining skin homeostasis. Thus, CD1d-restricted NKT cells can link innate and adaptive immunity, despite the small number of these cells in the skin. In addition, CD1d-restricted NKT cells can regulate immune responses by interacting with non-immune cells (i.e., fibroblasts and keratinocytes) and immune cells (i.e., Langerhans cells, dermal DCs, Breg cells, and B1 cells) during skin inflammation. Furthermore, staphylococcal superantigens (SsAgs), such as staphylococcal enterotoxin B (SEB), bind to both MHC II expressed on APC and TCR Vβ8 chain of CD1d-restricted NKT cells, consequently bridging interaction between APC and NKT cells *via* antigen-independent manner. Thus, TCR Vβ8-expressing NKT cells might be involved in regulating *S. aureus* pathogenesis in the skin even without glycolipid antigens. Moreover, upon cross-talk with various cell types, CD1d-restricted NKT cells produce soluble factors (e.g., IFNγ, IL2, IL4, IL13, TNFα, perforin, and granzymes), which are either protective or pathogenic in inflammatory skin diseases. AD, atopic dermatitis; ACD, allergic contact dermatitis; APC, antigen-presenting cells; β-GlcCer, β-D-glucopyranosylceramide; Breg cells, regulatory B cells; DCs, dendritic cells; LCs, Langerhans cells; SsAgs, staphylococcal superantigens.

CD1d-restricted NKT cells are critical immune mediators in regulating skin inflammatory responses (**Figure 1** and **Table 1**). Thus, modulating the effector functions of NKT cells may be explored to develop therapeutics for skin immune diseases. For example, the effects of NKT cell activation could be altered by the protocol employed to administer glycolipids [i.e., dosage (82), frequency (83), route (84, 85)]. Further, distinct types of NKT cell-stimulating glycolipids can contribute to the immune balance between Th1 and Th2 responses (86–88). Interestingly, it has been reported that the long-chain fatty acid palmitate (C16:0) directly activates iNKT cells to induce a decrease in IFNγ and IL4 (89), but an increase in IL10 production (90) *via* inositol-requiring enzyme 1α. Such iNKT cell-produced IL10 ultimately suppresses inflammatory responses, suggesting palmitate as a promising candidate to treat inflammatory skin diseases.

**Table 1:** Roles of CD1d-restricted NKT cells in various inflammatory skin diseases.

Diseases	NKT type	Subtype	Relativeproportion	Species	Cellular source	Cytokines/Signaling molecules		Functions	References
						Increase	Decrease		
AD	I	CD4^-^	**↓**	H	PBMC	‐	‐	‐	(10)
	I	DN	**↓**	H	PBMC	‐	‐	‐	(12)
	I	CD161^+^	**↓**	H	PBMC	‐	‐	‐	(13)
	I	CD4^+^	**↑**	H	PBMC	‐	‐	‐	(14)
	I	DN	**↓**	H	PBMC	IL4	IFNγ	‐	(15)
	I	‐	**↑**	H	PBMC, Skin	‐	‐	‐	(23)
	I	CXCR4^+^	**↑**	H	Skin	‐	‐	‐	(17)
	I	CXCR4^+^	**↑**	M	Skin	IFNγ, IL4, IL17	‐	Pathogenic	(17)
	I	DN	**↑**	M	Skin	IFNγ, IL2	‐	Protective	(19)
	I	DN	**=**	M	Spleen	IL4, IL10	IFNγ	Pathogenic	(22)
ACD	I	‐	**↑**	H	Skin	IFNγ, IL4	‐	‐	(38)
	I	CD4^+^, DN	**↑**	M	Spleen	IFNγ	‐	‐	(39)
	I	‐	**↑**	H	Skin	Perforin, Granzyme B, K	‐	‐	(41)
	I	‐	‐	M	‐	IL4, IL13	‐	Protective	(45)
	I, II	‐	‐	M	‐	‐	‐	Protective	(46)
	I	‐	‐	M	‐	‐	‐	Pathogenic	(51)
Psoriasis	I	‐	**↓**	H	‐	‐	‐	‐	(53)
	I	CD69^+^	**↓**	H	PBMC	IL4, IL17, GATA3, RORγt	‐	‐	(55)
	I	CD161^+^	**↑**	H	Skin	‐	‐	‐	(59)
	I	‐	**↑**	H	Skin	PKCζ	‐	‐	(61)
	I	CD161^+^	**↑**	H	Skin	IFNγ	‐	Pathogenic	(58)
UV-induced skin inflammation	I, II	‐	‐	M	‐	‐	‐	Pathogenic	(66)
	I	‐	‐	M	Lymph nodes	IL4	‐	Protective	(67)
	I	‐	‐	M	‐	‐	‐	Protective	(68)
	I, II	‐	‐	M	‐	‐	‐	Pathogenic	(68)
Scleroderma	I	‐	**↓**	H	PBMC	IL17	‐	‐	(75)
Alopecia areata	I	‐	**↑**	H	Skin	IL10	‐	Protective	(80)
Vitiligo	I	CD4^+^	**↓**	H	PBMC	‐	‐	‐	(81)
Skin wound healing	I	‐	‐	M	‐	‐	‐	Protective	(77–79)

I, type I; II, type II;‐, not evaluated; DN, double negative; ↑, increase; ↓, decrease; =, no change; H, human; M, mouse; PBMC, peripheral blood mononuclear cells.

From the perspective of developing topical therapeutics for skin diseases, the skin barrier remains a significant challenge. Thus, there is growing interest in designing safe and effective drug delivery systems. One example is nanocarriers such as liposomes and micelles to help increase the penetration of drugs through the skin barrier (91). In particular, palmitate-containing liposomes may provide significant therapeutic benefits to iNKT cell-mediated skin inflammation (92). In addition, as increased β-GlcCer accumulation by the reduction of GlcCer’ase activity affects NKT cell activation (63, 93), extracellular vesicle-based delivery of GlcCer’ase represents a promising therapeutic approach (94). Furthermore, the smaller the nanoparticles, the higher their drug delivery efficiency to the skin (95). Recently, we have demonstrated that nano-sized graphene oxide (nGO) mediates anti-inflammatory responses *via* conversion of iNKT cells toward a regulatory phenotype (96). Thus, nGO could be a promising strategy to modulate iNKT cells for suppressing inflammatory skin diseases.

As already noted, iNKT cells are functionally divided into several groups depending on the expression of transcription factors. Despite emerging evidence on distinct roles of iNKT cell subsets in various immune responses, little is known about their involvement in inflammatory skin diseases. Thus, it will be important to explore the precise immunoregulatory mechanisms of the skin resident iNKT cell subsets to develop better therapeutic agents for skin inflammation.

## Author contributions

SL has done the literature search. SL and SH wrote the first draft. SL, SH, HP, and LVK edited the manuscript. All authors contributed to the article and approved the submitted version.

## Funding

This work was supported by the Basic Science Research Program through the National Research Foundation of Korea (NRF) funded by the Ministry of Education (NRF-2021R1I1A1A01054418 to SL; NRF-2021R1I1A1A01051465 to HP; NRF-2022R1A2C1009590 to SH).

## Conflict of interest

LVK is a member of the scientific advisory board of Isu Abxis Co., Ltd. (South Korea).

The remaining authors declare that the research was conducted in the absence of any commercial or financial relationships that could be construed as a potential conflict of interest.

## Publisher’s note

All claims expressed in this article are solely those of the authors and do not necessarily represent those of their affiliated organizations, or those of the publisher, the editors and the reviewers. Any product that may be evaluated in this article, or claim that may be made by its manufacturer, is not guaranteed or endorsed by the publisher.
